# Deep learning based dual stage model for accurate nasogastric tube positioning in chest radiographs

**DOI:** 10.1038/s41598-025-98562-3

**Published:** 2025-04-25

**Authors:** Inseo Park, Gwiseong Moon, Ji Young Hong, Jeongwon Heo, Hongseok Ko, Doohee Lee, Yoon Kim, Woo Jin Kim, Hyun-Soo Choi, Kyoung Min Moon

**Affiliations:** 1 Department of Research and Development, ZIOVISION Co. Ltd., Chuncheon, 24341 Kangwon Republic of Korea; 2 Division of Pulmonary and Critical Care Medicine, Department of Medicine, Chuncheon Sacred Heart Hospital, Hallym University Medical Center, Chuncheon, 24253 Republic of Korea; 3 Department of Internal Medicine, Kangwon National University, Chuncheon, 24341 Republic of Korea; 4 Department of Radiology, Kangwon National University Hospital, Chuncheon, 24341 Republic of Korea; 5 Department of Computer Science and Engineering, College of IT, Kangwon National University, Chuncheon, 24341 Republic of Korea; 6 Department of Computer Science and Engineering, Seoul National University of Science and Technology, Seoul, 01811 Republic of Korea; 7 Division of Pulmonary and Allergy Medicine, Department of Internal Medicine, Chung-Ang University Hospital, Seoul, 06973 Republic of Korea; 8 Department of Radiology, Seoul National University Hospital, Seoul, 03080 Republic of Korea

**Keywords:** Computer science, Medical imaging

## Abstract

Accurate placement of nasogastric tubes (NGTs) is crucial for ensuring patient safety and effective treatment. Traditional methods relying on manual inspection are susceptible to human error, highlighting the need for innovative solutions. This study introduces a deep-learning model that enhances the detection and analysis of NGT positioning in chest radiographs. By integrating advanced segmentation and classification techniques, the model leverages the nnU-Net framework for segmenting critical regions and the ResNet50 architecture, pre-trained with MedCLIP, for classifying NGT placement. Trained on 1799 chest radiographs, the model demonstrates remarkable performance, achieving a Dice Similarity Coefficient of 65.35% for segmentation and an Area Under the Curve of 99.72% for classification. These results underscore its ability to accurately distinguish between correct and incorrect placements, outperforming traditional approaches. This method not only enhances diagnostic precision but also has the potential to streamline clinical workflows and improve patient care. A functional prototype of the model is accessible at https://ngtube.ziovision.ai.

## Introduction

Deep learning (DL) technologies, one of the hot topics within the field of machine learning, have demonstrated robust performance in data analysis, showcasing superior efficacy compared to conventional methods across a spectrum of research areas such as visual recognition, natural language processing, and sentiment analysis.^1^ In recent times, DL-based applications have emerged as a significant focus in the healthcare field, where they have exhibited notable successes.^2^ These trends have led to the employment of DL models for the automatic detection of abnormalities in tube positions, producing significant results.^3–7^ Specifically, the DenseNet architecture8 was used for the automated detection of nasogastric (NG) tubes misplaced in the respiratory tract on chest radiographs.^3^ Similarly, the Inception-v3 model9 was utilized to detect NG tube malposition on chest radiographs.7 Additionally, EfficientNet10 and U-Net11 (with EfficientNet backbone) architectures have been employed to identify the presence and position of central venous catheters, NG tubes, and endotracheal tubes on chest X-ray images.^4^ However, previous studies^3–11^ have predominantly concentrated on classification models, which posed limitations on explaining and visualizing the NG tube position through methods such as Grad-CAM. This limitation arises because classification models typically provide a probabilistic output indicating the presence or absence of a feature (e.g., a misplaced tube) without giving a detailed spatial representation of the tube’s position. Consequently, these earlier models presented challenges for clinical practitioners who needed to accurately determine whether the NG tube placement was complete (and thus safe for patients) or incomplete (posing safety risks to patients) in a clinical setting. To address these limitations, the authors have conducted a comprehensive deep-learning research endeavor aimed at enabling all healthcare providers, regardless of their radiological expertise, to intuitively assess the completeness (safety) or incompleteness (risk) of NG tube positions for patient care. This research involved not only segmenting the nasogastric(NG) tube locations directly but also performing multiple stages of concatenation and classification. By integrating segmentation techniques, the research advances the field by providing more detailed visualizations and explanations of the tube positions, which are crucial for clinical decision-making.

The segmentation approach allows the model to delineate the exact location and trajectory of the NG tube within the chest radiograph, thereby offering a visual guide that clinicians can easily interpret. This method enhances the interpretability of the model’s predictions, making them more practical for real-world clinical applications. Our main contributions can be summarized as follows:

- We collected and curated a comprehensive dataset of chest X-rays from patients, specifically annotated for NG tube positioning. This ensured data diversity and quality, enhancing the generalizability and robustness of the model.
- We developed a novel dual-stage model combining nnU-Net for segmentation and ResNet50, pre-trained with MedCLIP, for classification. The model achieved significant performance metrics, including a Dice Similarity Coefficient of 65.35% and an AUC of 99.72%, demonstrating high accuracy and reliability.
- We created a functional prototype that can be integrated into clinical workflows, providing real-time assistance for NG tube positioning in chest radiographs. The prototype was validated in a clinical setting, highlighting its potential for improving patient safety and treatment effectiveness. The prototype is available at https://ngtube.ziovision.ai.

## Materials and methods

### Ethics approval and consent to participate

This retrospective study was approved by the Institutional Review Board (IRB) of Chuncheon Sacred Hospital, which waived the requirement for informed consent (approval number: CHUNCHEON 2023-12-006) and all methods were performed following applicable guidelines and regulations

### Study population

In this study, We curated a dataset of 2,627 anonymized patient X-ray images from three healthcare institutions: Hallym University Sacred Heart Hospital, Gangneung Asan Hospital, and Kangwon National University Hospital, collected between April 2011 and January 2023. Specifically, the dataset includes 263 images from Hallym University Sacred Heart Hospital, 1,986 images from Gangneung Asan Hospital, and 378 images from Kangwon National University Hospital. Data from Hallym University Sacred Heart Hospital and Gangneung Asan Hospital were used as our internal dataset, with 450 images randomly selected as the test set. The data from Kangwon National University Hospital was exclusively used for external validation. All images were meticulously extracted and stored in DICOM format using an advanced Picture Archiving and Communication System (PACS). For a more detailed understanding of the patient characteristics within the dataset, a straightforward overview is provided in Table 1

**Table 1:** Data characteristics including patient demographics, image properties, and data sources. Values with a plus/minus sign represent the means ± standard deviation. Note that the values of age and sex in the external testing set are not provided because they were de-identified before data collection. $\chi ^{2}$ test was used for *P*-value of categorical variables such as label, sex, and manufacturer. Analysis of variance (marked as $\dagger$) was used for age.

Variable	Training & validation dataset	Internal testing dataset	External testing dataset	*P*-value
Total (*n*)	1799	450	378	
Label (*n, %*)				0.12
Complete	1566 (87.1)	391 (86.9)	314 (83.1)	
Incomplete	233 (12.9)	59 (13.1)	64 (16.9)	
Sex (*n, %*)				0.77
Male	974 (54.1)	275 (61.1)	–	
Female	600 (33.4)	175 (38.9)	–	
Unknown	225 (12.5)	–	–	
Age (years, mean ± SD)	$77.50 \pm 11.20$	$77.51 \pm 10.96$	-	$0.98^{\dagger }$
Manufacturer (*n, %*)				0.0001
Canon	608 (33.8)	176 (39.1)	–	
DongKang	582 (32.3)	166 (36.9)	–	
DK Medical Systems	156 (8.7)	44 (9.8)	–	
Samsung	66 (3.7)	21 (4.7)	172 (45.5)	
SIEMENS	–	–	158 (41.8)	
Other	387 (21.5)	43 (9.6)	48 (12.7)	

### Ground truths

To obtain the labeled data, clinical experts annotated the regular insertion of the NG tube as ‘complete’ and the abnormal state as ‘incomplete’ in the opinion of 4 qualified specialists (MKM, KHG, HJY, and HJW). Three respiratory medicine experts (MKM, HJY, and HJW) with over 10 years of clinical experience, along with one radiology expert (KHS), collected a Chest X-ray dataset of 2249 cases labeled for NG tube placement. All cases were classified as either ‘complete’ or ‘incomplete.’ Clinically, NG tube positions where patients could receive medication or proceed with enteral feeding were categorized as ‘complete’ and defined as normal NG tube positions. Cases, where NG tube use for medication or feeding was not possible, were classified as ‘incomplete’ and defined as abnormal NG tube position. The tip position was measured at least 3 cm distal to the diaphragm on Chest X-ray PA or AP views for cases where the NG tube did not enter the airway. In situations where one clinical expert found it difficult to classify as ‘complete’ or ‘incomplete,’ all four experts gathered together to review the NG tube position on the Chest X-ray simultaneously. If they all agreed, the case was labeled as ‘complete’ and considered to have a normal NG tube position.^12^ Through this process, we used 1957 complete samples and 292 incomplete samples for model training and testing.

**Fig. 1:**
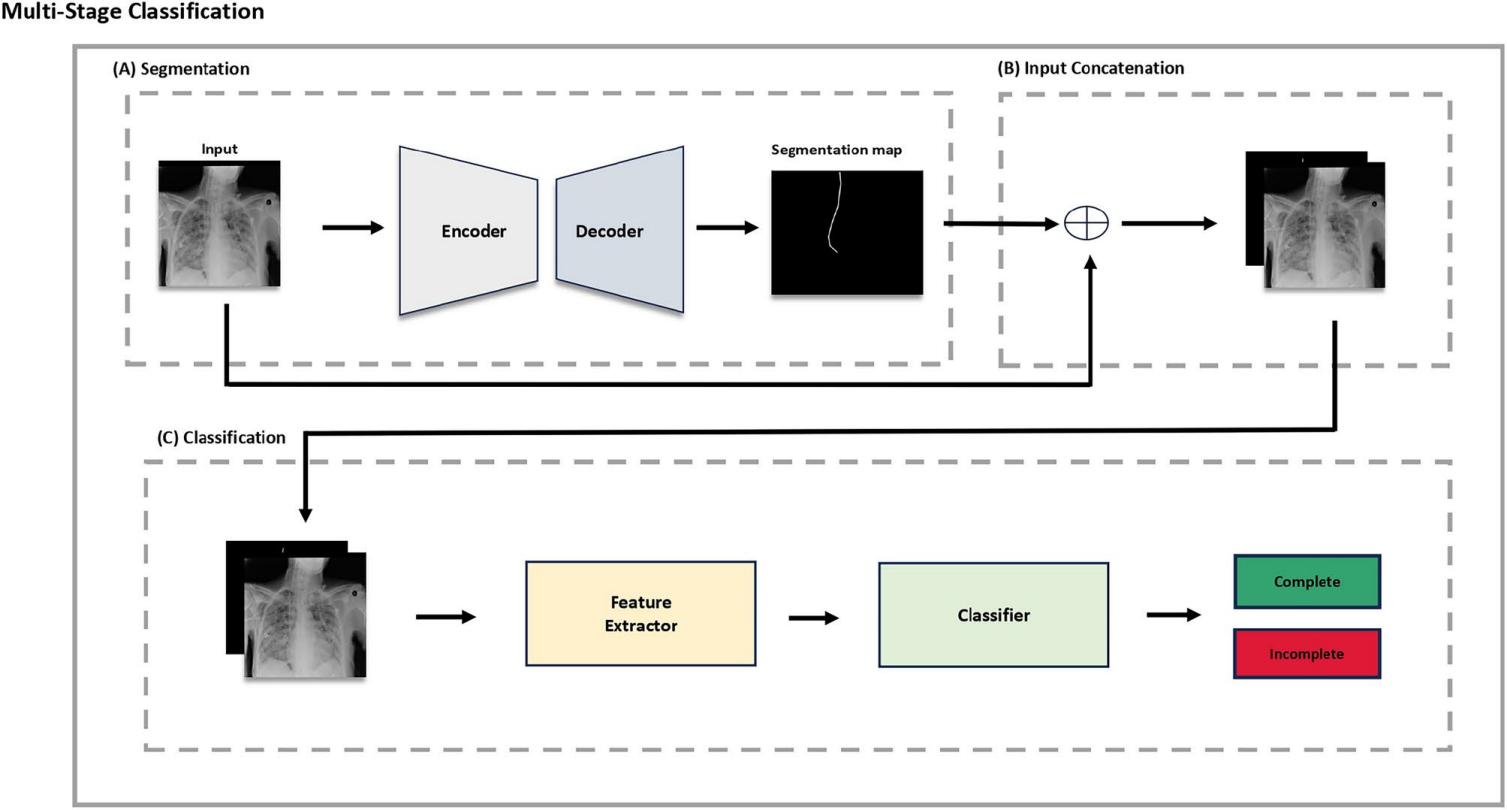
Workflow of multi-stage classification on chest X-ray images: **(a)** Segmentation stage, **(b)** input concatenation stage, **(c)** classification stage.

### Workflow overview

In a clinical setting, doctors find a NG tube in a chest X-ray image and then use it and surrounding information to identify abnormalities in the tube’s location. We intend to implement similar methodologies. Our algorithm expands in three key stages: segmentation, concatenation, and classification, each contributing to the comprehensive workflow illustrated in Fig. 1. In the segmentation stage, a deep learning-based segmentation model is employed to delineate NG tube masks from X-ray images, showcasing the model’s proficiency in segmenting relevant structures. The generated masks from the segmentation model are combined with their corresponding X-ray images. This approach facilitates the shape information of the images by feeding the combined images to a model used in the next stage. Finally, in the classification stage, a specialized model suggests whether a NG tube is fully and safely inserted. The harmonious integration of these stages culminates in a robust and effective approach for NG tube analysis from X-ray images.

### Segmentation

The proposed methodology begins with the primary objective of segmenting NG tube lines, which imparts shape awareness to the subsequent classification model. For this task, we introduced a dedicated segmentation model denoted as $f_\theta$, specifically designed to identify NG tube masks, including lines and tips within X-ray images. The model $f_\theta$ operates to generate masks of a line $m_l \in \{0, 1\}^{H \times W}$ and a tip $m_t \in \{0, 1\}^{H \times W}$ from a pre-processed and normalized image $x$ with respect to sample index $i$:

1 $$\begin{aligned} \textbf{m}^{s \in \{l, t\}}_i = \textrm{argmax}(\Phi (f_\theta (\textbf{x}_i))), \end{aligned}$$

where $\Phi$ denotes the post-processing of nnU-Net^13^. This process ensures the extraction of appropriate information regarding the NG tube lines, setting the stage for enhanced shape awareness in preparation for the subsequent stages of our method.

We employed nnU-Net, a state-of-the-art framework based on U-Net architecture for biomedical segmentation, as our segmentation model $f_\theta$ to achieve precise NG tube detection. To enhance the accuracy of our segmentation model, we strategically employed a pre-training technique. Specifically, the model $f_\theta$ underwent pre-training on the RANZCR CLiP dataset.^14^

### Classification

While traditional convolutional neural networks often rely on predictions based on individual intact images, recent studies have highlighted the advantages of leveraging the outcomes of neural networks as inputs for subsequent neural networks, resulting in improved performance^15–17^. Inspired by these findings, our approach incorporates this concept by employing a concatenation operation ($\odot$) to aggregate images and masks, thereby creating a more informative input.

2 $$\begin{aligned} \textbf{z}_i = \textbf{x}_i \odot \textbf{m}^{s \in \{l, t\}}_i. \end{aligned}$$

where $x_i$ is the X-ray image and $m_{s \in \{l, t\}}$ is the segmented mask. We believe that this concatenated input $z$ significantly improves the model’s understanding of anatomical structures and NG tube masks in X-ray images. In the classification stage, we utilized this improved input to train our model, denoted as $g_\theta$, to accurately predict abnormalities in the position of the NG tube. The concatenated results $z$ were fed into the model $g_\theta$, which then calculated the probabilities $p$ of NG tube malposition as $p_i = \sigma (g_\theta (z_i))$, with $\sigma$ being the activation function. These probabilities were optimized using the cross-entropy loss function, enabling the model to classify positions as either’satisfactory’ or’malposition’.

To improve the model’s generalization and robustness, we applied a set of augmentations in the training pipeline, including horizontal flipping, random brightness and contrast adjustments, and geometric transformations such as shift, scale, and rotation with border mode handling. These enhancements were designed to introduce variations in the data set, reducing possible biases and enhancing the model’s ability to learn relevant features from diverse input distributions.

We used ResNet-based architectures^18^, which are the most widely used neural networks in image analysis, as the model $g_\theta$. For more accurate performance, we also used a pre-trained MedCLIP model^19^, a state-of-the-art medical vision text pre-training model, for the classification network $g_\theta$.

Furthermore, to improve the interpretability of our model and to understand the decision-making process behind its predictions, we incorporated Gradient-weighted Class Activation Mapping (Grad-CAM)^20^. Grad-CAM provides a visual explanation for the model’s decisions, highlighting the specific areas in the X-ray images that the model focuses on when determining NG tube positioning. This approach not only helps validate the accuracy and reliability of the model, but also helps identify potential areas for further training or refinement, thus contributing to the continuous improvement of the model’s performance.

### Implementation details

We employed the PyTorch framework to implement all networks and experimental settings. Our segmentation model underwent training via a five-fold cross-validation to enhance robustness, and an ensemble strategy was employed to ensure precise segmentation results. Due to significant differences in image sizes, we set the input patch size to 512 $\times$ 512. We apply similar implementation details of the nnU-Net, such as the optimizer and data augmentation techniques. In the classification stage, all networks were trained on a single NVIDIA RTX 3090 GPU, and all network parameters were optimized by SGD optimizer on stratified five-fold cross-validation. We set a learning rate of 0.0005 for the feature extractors in the classification model, which is ten times lower than that of the classifiers that consist of a single MLP layer. All networks are trained for 100 epochs with a batch size of 16, and we initialize the feature extractor’s weights by MedClip’s ResNet-50 and classifiers with random weights. All models’ inputs were uniformly resized to 512 $\times$ 512, and a random horizontal flip was applied with a probability of 0.5 for training. We split the training dataset into training (80%) and validation (20%) datasets, and all hyperparameters were tuned in the validation phase.

## Results

### Experimental settings

1,799 and 450 images were used in the training and testing datasets, respectively. During the segmentation phase, the training dataset was used as a 5-fold cross-validation (stratified cross-validation in the classification stage), and the testing set was used to report the model’s performance. We also used 378 images from Kangwon National University Hospital for external validation to further assess the robustness and generalizability of our classification model

### Performance metrics

As our method includes two models with different abilities (*i.e.* segmentation, classification), we reported six metrics to evaluate each of them. To evaluate the segmentation model, we first employed Dice and Jaccard coefficient metrics, which present similarities between predictions and ground truth. We formulate the metrics by true positive, false positive, and false negative denoted TP, FP, and FN, respectively.

3 $$\begin{aligned} \textrm{Dice}= &   \frac{2TP}{2TP + FP + FN}, \end{aligned}$$

4 $$\begin{aligned} \textrm{Jaccard}= &   \frac{TP}{TP + FP + FN}. \end{aligned}$$

Considering imbalanced class distribution, we also reported macro F1-score, balanced accuracy (B-ACC), area under the receiver operating characteristics (AUC-ROC), and area under the precision-recall curve (AUC-PR) for evaluation of the classification model. F1-score and B-ACC are formulated as below:

5 $$\begin{aligned} \operatorname {F1-score}= &   2 \times \frac{\textrm{Precision} \times \textrm{Recall}}{\textrm{Precision} + \textrm{Recall}}, \end{aligned}$$

6 $$\begin{aligned} \operatorname {B-ACC}= &   \frac{\textrm{Sensitivity} + \textrm{Specificity}}{2}. \end{aligned}$$

### Segmentation performance

We applied the Exponential Moving Average (EMA) to track performance and find the best model during the validation stage. To make robust and precise predictions, we ensemble the logit of the trained models in five folds to evaluate the testing dataset. The segmentation model achieved a Dice of 65.35% and a Jaccard of 57.49% on the testing dataset, as shown in Table 2

**Table 2:** 5-Fold cross-validation results for NGT segmentation on the testing dataset.

	Tube	Tip	Mean
Dice, mean ± SD	91.52 ± 0.14	39.16 ± 0.40	65.35 ± 0.25
Jaccard, mean ± SD	85.63 ± 0.22	29.37 ± 0.31	57.49 ± 0.21

### Classification performance

As seen in Table 3, methods utilizing the concatenated input with segmented masks ($\textbf{m}^l$ or $\textbf{m}^t$) consistently enhanced the model’s classification ability on all metrics: F1-score, B-ACC, AUC-ROC, and AUC-PR. Compared with the method using only original images, the combination of image and line mask ($\textbf{x} + \textbf{m}^l$) showed increased performance by 1.74$\%$, 3.71$\%$, 1.79$\%$, and 2.09$\%$, respectively. Similarly, the combination of the image and all masks ($\textbf{x} + \textbf{m}^l + \textbf{m}^t$) showed increased performance by 0.66$\%$, 2.14$\%$, 1.55$\%$, and 1.5$\%$. Although all combinations of the masks helped improve the model’s performance, leveraging all masks ($\textbf{x} + \textbf{m}^l + \textbf{m}^t$) showed lower performance by 1.08$\%$, 1.57$\%$, 0.24$\%$, and 0.59$\%$ than using line masks alone. From these observations, we believe that the concatenated input with only line masks is more suitable than others. As shown in Table 4, ResNet-50 outperformed other architectures, except MedCLIP pre-trained ResNet-50. From these observations, we can confirm that ResNet-50 is more suitable in our experiment setting. Next, the MedCLIP pre-trained ResNet-50 showed improved performance than the vanilla ResNet-50 by 2.11$\%$ 1.79$\%$ 0.38$\%$ and 0.85$\%$, respectively. These results suggest the possibility of detecting incorrect insertions of NG tube through deep learning-based methods. In addition, the external validation indicates F1-score, B-ACC, AUC-ROC, and AUC-PR values of 78.99%, 83.94%, 92.27%, and 86.78%, respectively. This not only reinforces the model’s capability in a broader clinical context but also highlights its potential in diverse healthcare settings.

**Fig. 2:**
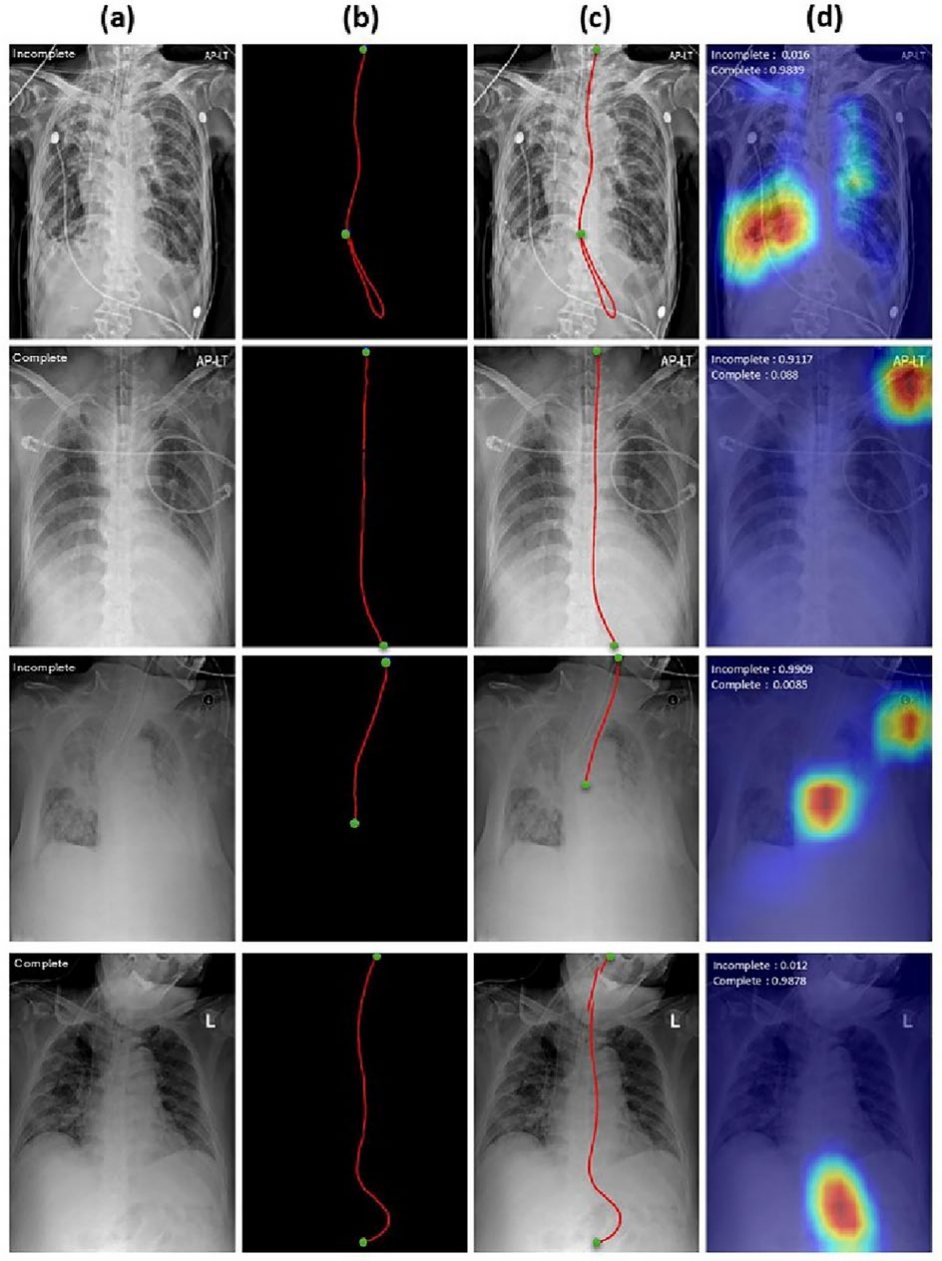
Visualization results of prediction masks and class activation map (Grad-CAM) on external validation datasets. Prediction masks are extracted from nnU-Net and Grad-CAM results are from MedCLIP pre-trained ResNet-50. (**a**) X-ray images, (**b**) label of the segmentation model, (**c**) results with the sum of (**a**) and prediction of the segmentation, (**d**) Grad-CAM results. The top two low are the incorrectly predicted cases(incomplete, complete) and the bottom two low(incomplete, complete) are the correctly predicted cases.

### Visualization of model predictions using grad-CAM

In our analysis, the utilization of Grad-CAMs revealed both the strengths and limitations of our model in discerning the placement of NG tubes within radiographs. The model accurately identified properly positioned tubes as shown by targeted Grad-CAM activations. However, our model faced challenges in identifying malpositioned tubes, often failing to detect tubes that were not correctly placed. Additionally, Grad-CAM occasionally highlighted areas unrelated to the tube, such as irrelevant text or peripheral regions, suggesting that the model might be influenced by non-essential features. These results are illustrated in Fig. 2.

**Table 3:** Performances for combinations of images and masks. All experiments are based on ResNet-50 with MedCLIP. Results are reported as mean ± standard deviation (SD).

Methods	Macro F1-score	B-ACC	AUC-ROC	AUC-PR
*x* ,mean ± SD	96.09±1.02	95.12±2.17	98.17±1.04	97.80±0.61
$x + m^l$ , mean ± SD	97.83±1.09	98.83±0.66	99.96±0.03	99.89±0.11
$x + m^l + m^t$ , mean ± SD	96.75±1.31	97.26±0.31	99.72±0.14	99.30±0.33

**Table 4:** Stratified 5-fold cross-validation results for NGT positioning classification on the testing and external datasets. Results are reported as mean ± standard deviation (SD).

Methods	Macro F1-score	B-ACC	AUC-ROC	AUC-PR
ResNet-18 ($x + m^l$)	94.36±7.20	96.27±4.64	97.98±3.85	93.61±12.09
ResNet-34 ($x + m^l$)	93.95±7.51	95.44±5.09	97.78±3.62	93.38±11.82
ResNet-50 ($x + m^l$)	95.72±3.98	97.04±1.61	99.57±0.35	99.04±0.85
ResNet-50 (pre-trained w/ MedCLIP) ($x + m^l$)	97.83±1.09	98.83±0.66	99.96±0.03	99.89±0.11
ResNet-50 (pre-trained w/ MedCLIP) ($x + m^l$) (external)	78.99±2.37	83.94±3.23	92.27±2.59	86.78±3.62

**Fig. 3:**
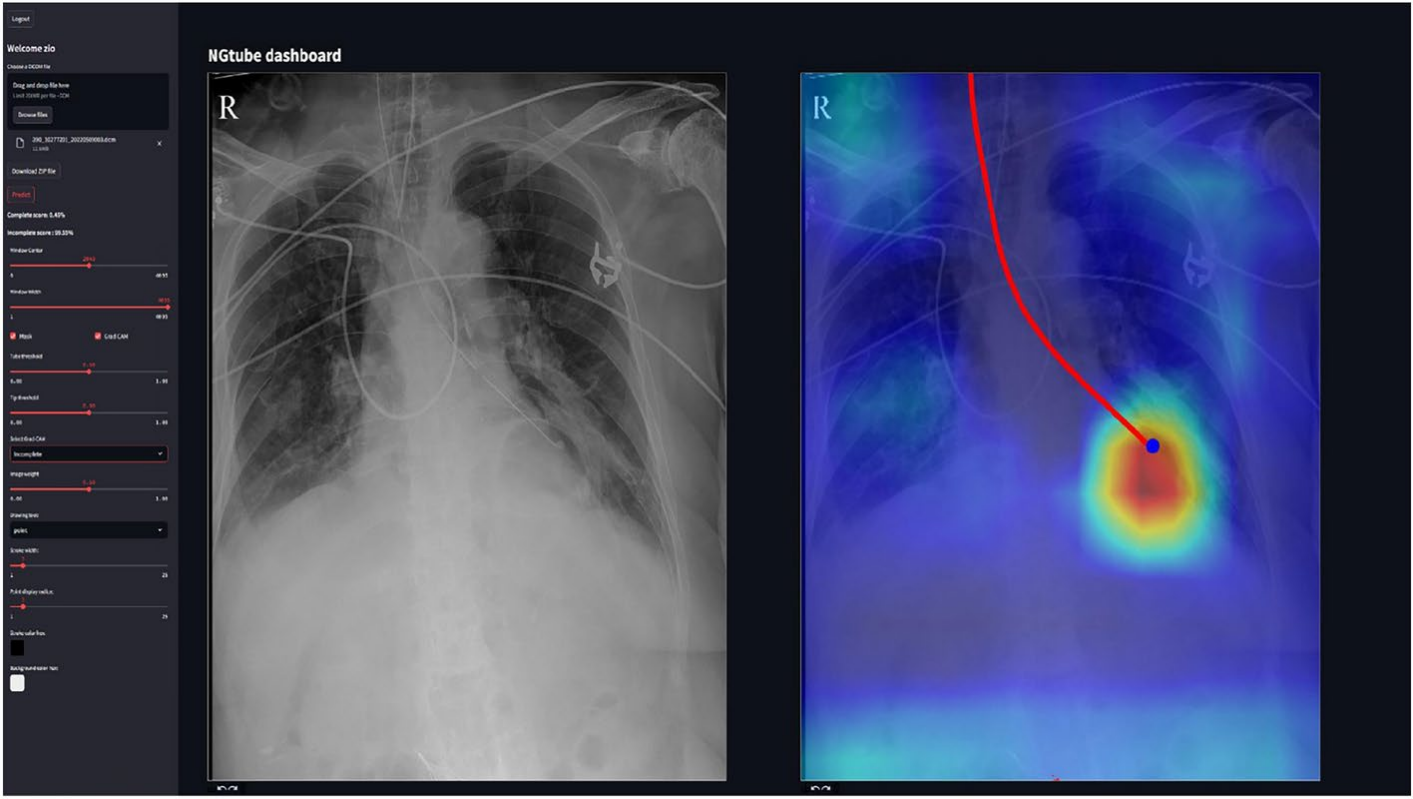
Prototype user interface for nasogastric tube positioning analysis.

### Prototype implementation

To address the clinical applicability of our research, we have implemented a functional prototype of our solution. This prototype is designed to be integrated into clinical workflows, providing real-time assistance for NG tube positioning in chest radiographs. The prototype has been developed to facilitate seamless adoption in healthcare settings, offering intuitive user interfaces and robust performance. An example of the prototype user interface is shown in Fig. 3. The prototype is available at https://ngtube.ziovision.ai

## Discussion

In this paper, we present a deep learning model for the segmentation and classification of NG tube in chest radiographs, achieving a Dice Similarity Coefficient of 65.35±0.25, an AUC of 99.72, and an AUPRC of 99.30. This performance highlights the effectiveness of our model. Our model integrates segmentation and classification to improve the analysis of NG tube in chest radiographs. We used nnU-Net for segmentation and Resnet50 pre-trained with MedClip for the classification task. This pre-training enables the model to interpret and analyze the intricate patterns in chest X-rays, improving its accuracy in confirming the positioning of the NG tube. A key improvement of our model is the combination of the segmentation mask generated by nnU-Net with the chest X-ray input to the Resnet50 classification model. This approach improves the accuracy of the model by incorporating additional contextual information from the segmentation data, thereby increasing the reliability of NG tube positioning.

Several studies have proposed deep learning-based methods for detecting NG tube malposition in chest radiographs.^5^ used 5475 radiographs to train and test CNN models, with the pre-trained Inception V3 achieving an AUC of 0.87.^3^ trained a DenseNet-based model on 4693 radiographs, showing high accuracy with AUCs of 0.90 and 0.92 for internal and external validation.^6^ introduced a CNN with a unique pre-training strategy, improving AUC from 0.56 to 0.76 and accuracy from 0.69 to 0.79 using 175 radiographs.^7^ trained a model on 7081 radiographs, achieving an AUC of 0.90 and improving agreement between junior physicians and radiologists. These studies highlight the potential of deep learning in improving NG tube placement accuracy and patient safety.

Most previous studies have primarily focused on classifying NG tube malposition. However, to enhance the model’s reliability and assist medical professionals in making informed decisions, it is crucial to not only provide information about malposition but also localize the NG tube. We addressed this need by integrating segmentation with classification. As a result, we not only obtained segmentation information but also achieved improved classification performance through this integration.

The utilization of Grad-CAMs provided valuable insights into the model’s decision-making process. Grad-CAM activations demonstrated the model’s capability to correctly identify properly positioned tubes. However, challenges were observed in accurately identifying incomplete tubes, with some instances of the model being influenced by non-essential features. This highlights areas for further refinement, particularly in improving the model’s sensitivity to incomplete tubes.

Our study has several limitations. First, the segmentation performance for the NG tube tip is suboptimal, likely due to inconsistencies in its definition. Initially, we considered both the start and end of the tube as the tip, but in reality, the tip refers to the most distal visible point in X-ray images. This broad definition may have introduced ambiguity, making accurate segmentation more difficult. To address this issue, it is necessary to refine the annotation process by labeling only the clearly visible distal tip.

Second, the interpretability of the model’s decision-making process remains a challenge. While Grad-CAM was used to visualize the model’s attention, it sometimes highlights irrelevant regions, such as textual artifacts or non-essential structures in X-ray images. To address this, developing quantitative evaluation methods and considering strategies like bounding-box constraints or an auxiliary attention-gating network could help improve interpretability and reduce irrelevant focus. Additionally, alternative interpretability techniques such as self-attention visualization or feature attribution analysis could provide complementary insights.

Third,The difference in X-ray equipment across datasets may have contributed to the model’s performance degradation in the external test set. As shown in Table 1, the distribution of imaging manufacturers varies significantly between the training, validation, and external test datasets. This variation in imaging devices introduces a domain shift, which can negatively affect model generalizability. Previous research^7^ has demonstrated that model performance can be biased by differences in imaging equipment, further supporting the need to address this issue. Moreover, differences in imaging equipment can introduce bias not only in model performance but also in its interpretation of X-ray images. Variations in imaging conditions, such as contrast, resolution, and noise levels, may cause the model to learn spurious correlations rather than clinically relevant features, leading to systematic errors in decision-making. To mitigate this problem, future work should consider domain adaptation techniques or dataset augmentation strategies that account for variability in imaging sources, thereby reducing both performance degradation and interpretational bias.

Fourth, class imbalance in our dataset presents a significant challenge. The proportion of malpositioned NG tube cases is significantly lower than that of correctly positioned tubes, with an approximate ratio of 7:1. This imbalance can lead to biased model predictions, particularly underrepresenting the minority class. Various approaches have been explored to address this issue, including uncertainty calibration, class-adaptive network calibration, and methods to correct overconfidence in dynamic neural networks. These techniques aim to enhance model reliability and improve performance in imbalanced settings. Future research should focus on developing more refined strategies to address class imbalance in medical imaging datasets and optimizing methods to improve model generalization in such challenging scenarios.

Fifth, our dual-stage model, which combines nnU-Net for segmentation and ResNet50 for classification, has demonstrated promising performance. nnU-Net was chosen for its automated preprocessing and optimization capabilities, providing strong baseline performance across various medical imaging tasks. However, future research should explore the potential benefits of evaluating the latest segmentation mode^21–25^ for further performance improvements. Additionally, integrating hybrid approaches that combine deep learning with traditional image processing techniques, such as edge detection or contour-based refinement, may further enhance segmentation accuracy.

Sixth, while our study focused on NG tube positioning, the proposed dual-stage approach can be extended to other types of lines and tubes, such as central venous catheters or endotracheal tubes. Given the structural similarities among these medical devices, our segmentation-classification framework could be adapted to assist in their localization and classification. Future work should explore this broader applicability, which could enhance the general utility of automated medical device detection in radiology.

To explore the clinical applicability of our research, we have developed a functional prototype available at https://ngtube.ziovision.ai. This prototype demonstrates the feasibility of automated NG tube positioning assistance and has the potential to improve accuracy, reduce interpretation time, and enhance clinical workflow efficiency. However, real-world deployment would require addressing key challenges such as PACS integration, computational efficiency, and data security. Future research should focus on optimizing model inference speed and developing seamless PACS connectivity to facilitate broader adoption in clinical settings.

## Conclusion

Our study presents a novel approach for NG tube positioning, significantly improving the detection accuracy in chest radiographs through advanced segmentation and classification techniques. The developed model offers a valuable tool for healthcare professionals, contributing to improved patient care. Future research will explore the application of Vision Transformer models to capture global image characteristics and expand the dataset with advanced cross-validation methods to further enhance the model’s generalizability and reliability.

## Data Availability

Data are available upon request by the corresponding author along with improvement of the data review board.

## Code availability

Code is available upon request by the corresponding author.
